# Complete Genome Sequence of Vibrio natriegens Phage Phriendly

**DOI:** 10.1128/MRA.01096-19

**Published:** 2019-10-03

**Authors:** James Clark, Adey Awah, Russell Moreland, Mei Liu, Jason J. Gill, Jolene Ramsey

**Affiliations:** a Center for Phage Technology, Texas A&M University, College Station, Texas, USA; DOE Joint Genome Institute

## Abstract

Vibrio natriegens is a fast-growing marine bacterium of interest for biotechnology applications. Here, we describe the isolation and genome annotation of *V. natriegens* podophage Phriendly. Among the 72 predicted coding genes and 1 tRNA, Phriendly’s 50,218-bp genome has low sequence identity to known phages.

## ANNOUNCEMENT

Vibrio natriegens, formerly known as Beneckea natriegens and Pseudomonas natriegens, is a nonpathogenic marine organism found commonly in brackish coastal waters (1). This organism is a Gram-negative curved rod and a moderate halophile of interest for biotechnological applications, partly owing to its rapid doubling time, reported as <10 min (2, 3). The isolation and genome annotation of Phriendly, a novel *V. natriegens* podophage, are described in this report.

Bacteriophage Phriendly was isolated from a filtered (0.2-μm pore size) salt marsh water sample collected near the shore of South Padre Island, TX. Phriendly was plaque purified three times; plaque formation in softagar overlays was observed on *V. natriegens* strain ATCC 14048 grown aerobically on LB (BD) at 37°C (4). Phage morphology was determined by viewing 2% (wt/vol) uranyl acetate negatively stained samples via transmission electron microscopy at the Texas A&M Microscopy and Imaging Center (5). Phage genomic DNA was purified as described by Summer, with the shotgun library preparation protocol (6). Samples were prepared for the Illumina MiSeq platform using the Illumina Nano low-throughput kit, and paired-end 250-bp reads were sequenced using v2 500-cycle chemistry. The 419,134 total sequence reads were quality controlled using FastQC (www.bioinformatics.babraham.ac.uk/projects/fastqc). Sequence reads were then trimmed using the FASTX-Toolkit v0.0.14 (http://hannonlab.cshl.edu/fastx_toolkit/). Genome assembly was performed using SPAdes v3.5.0, with default parameters (7). A single contig with 66.7-fold coverage was obtained and confirmed to be complete by Sanger sequencing of a PCR product amplified off the contig ends (forward primer, 5′-AAGCTCGTGTGGCTGTTAAA-3′; reverse primer, 5′-TGGTTCATCAGAGCCACTAAAC-3′). Phage termini were predicted from raw sequencing reads using PhageTerm (8). Gene calling was performed with Glimmer v3.0 and MetaGeneAnnotator v1.0 (9, 10). TransTermHP v2.09 provided prediction of Rho-independent termination sites (11). tRNA genes were detected using ARAGORN v2.36 (12). Functional predictions relied primarily on conserved domain analyses from InterProScan v5.33-72 and BLAST v2.2.31 (13, 14). The BLAST analysis used a 0.001 maximum expectation value cutoff to search the NCBI nonredundant (February 2019 version) and UniProtKB Swiss-Prot/TrEMBL (release 2018_11) databases (15). Transmembrane domain prediction was done with TMHMM v2.0 (16). The LipoP v1.0 tool analyzed lipoylation signals (17). Structural predictions were done with the HHSuite v3.0 tool HHpred (multiple-sequence alignment [MSA] generation with HHblits using the ummiclus30_2018_08 database and modeling with the PDB_mmCIF70 database) (18). Genome-wide sequence comparison to top BLAST nucleotide hits was calculated using the progressiveMauve v2.4.0 algorithm (19). All tools (except HHpred) were accessed and run with default parameters (unless otherwise stated) at the Center for Phage Technology Galaxy instance and annotation performed in Web Apollo (both hosted at https://cpt.tamu.edu/galaxy-pub/) (20, 21).

The 50,218-bp genome of podophage Phriendly has a G+C content of 41%. The genome was reopened to start with the direct terminal repeat predicted by PhageTerm, similar to T7-like phages. One tRNA gene and 72 protein-coding genes were predicted, with a coding density of 93%. Comparative genomics revealed that Phriendly has <5% nucleotide identity to known phages. In total, only 26 genes were assigned a putative function; among them is the predicted unimolecular spanin (NCBI accession number QEG09222) needed for lysis.

### Data availability.

The genome sequence and associated data for phage Phriendly were deposited under GenBank accession number MN062185, BioProject accession number PRJNA222858, SRA accession number SRR8892141, and BioSample accession number SAMN11408656.
